# Effects of leukoreduction and storage duration on whole blood hemostatic function: a prospective *ex vivo* observational study

**DOI:** 10.3389/fcell.2026.1765877

**Published:** 2026-03-27

**Authors:** Yingyu He, Xiaojie Wei, Yingkai Xu, Zihan Yuan, Junying Li, Changxin Qi, Fangxiong Cheng, Wanbing Liu, Dan Shu, Lei Liu

**Affiliations:** 1 Department of Transfusion Medicine, General Hospital of Central Theater Command, Wuhan, Hubei, China; 2 Department of Transfusion, Wuhan Fourth Hospital, Wuhan, Hubei, China; 3 Department of Laboratory Medicine, 988th Hospital of Joint Logistics Support Force of CPLA, Zhengzhou, Henan, China; 4 Medical College, Wuhan University of Science and Technology, Wuhan, Hubei, China; 5 Institute of Pharmaceutical Innovation, Hubei Province Key Laboratory of Occupational Hazard Identification and Control, School of Medicine, Wuhan University of Science and Technology, Wuhan, Hubei, China

**Keywords:** hemostatic resuscitation, leukoreduction, machine learning, platelet dysfunction, whole blood storage

## Abstract

**Background:**

Whole blood (WB) resuscitation is a critical component of trauma care. Leukoreduction (LR) is commonly employed to mitigate transfusion-related risks. We aimed to understand the effects of LR on the functionality and metabolism of various components in refrigerated WB and provide new strategies for WB application in trauma treatment.

**Methods:**

Ten bags of WB (400 mL ± 10%) were split into paired samples with and without LR and stored at 4°C ± 2 °C for 35 days. Storage-lesion tests were performed every 7 days from the day of blood collection. Descriptive heatmaps and weighted gene co-expression network analysis (WGCNA) were used to characterize global patterns of indicator change, while exploratory mediation models and penalized regression (LASSO) were applied to investigate pathways and rank variables associated with clot strength.

**Results:**

Descriptive heatmaps and exploratory WGCNA analyses suggested two distinct temporal patterns: platelet-related and viscoelastic indicators decreased progressively, whereas metabolic, hemolytic, and electrolyte indicators increased over time. On Day 0 (immediately after leukoreduction), leukoreduced whole blood (LR-WB) had a markedly lower platelet count than paired nonleukoreduced whole blood group (NLR-WB) (23.40 ± 2.00 vs. 185.20 ± 2.93 *10^9^/L; *p* < 0.05), accompanied by reduced clot strength and impaired kinetics (MA 33.6 ± 9.82 vs. 61.09 ± 2.97 mm; K 5.20 ± 1.37 vs. 1.70 ± 0.35 min; Angle 45.83 ± 8.82 vs. 65.68° ± 4.33°; all *p* < 0.05). These between-group differences persisted across storage. Fibrinogen concentration remained comparable between two groups, but kinetic indicators (K, Angle) were consistently lower in LR-WB. In exploratory mediation models, platelet count, mean platelet volume, K, and Angle accounted for most of the model-based associations of LR and storage duration with MA. Penalized regression (LASSO) further highlighted blood cells’ (RBCs’) phosphatidylserine (PS) exposure and hemoglobin (FHb) as late-storage features associated with lower MA within this experimental dataset.

**Conclusion:**

LR was associated with early platelet-driven hemostatic impairment, and storage time appeared to progressively shift injury mechanisms toward RBC membrane instability, hemolysis, and metabolic stress. RBCs’ PS exposure and FHb can serve as candidate indicators associated with clot strength.

## 1 Introduction

Whole blood (WB) transfusion has re-emerged over the past decade as a frontline strategy in military damage-control resuscitation, pre-hospital trauma care, and resource-limited environments. Beyond these environments, low-titer group O cold-stored whole blood (LTOWB) has also been adopted in civilian trauma centers for routine hemorrhagic resuscitation (Ghaedi et al., 2025). Clinical use and case series have additionally been reported in peripartum hemorrhage and in the management of acute blood loss in neonates and preterm infants (Bahr et al., 2019; Carr et al., 2022; Carr et al., 2024). Compared with component therapy, WB provides a physiologic ratio of red blood cells (RBCs), plasma, and platelets in a single unit, thereby simplifying logistics and accelerating correction of hemorrhagic shock (Gurney et al., 2022; Coulthard et al., 2024). Reviews of prehospital WB use have identified the need for routine leukoreduction (LR) as an unresolved issue (Ghaedi et al., 2025). This has made the necessity of LR in WB, and its potential trade-offs, a continuing subject of debate.

LR is no longer viewed solely as a measure to curb febrile non-hemolytic reactions or alloimmunization. Donor leukocytes and their cytokine cargo can influence transfusion-related immune responses, contributing to both immunosuppression and pro-inflammatory injury (Garraud et al., 2016; Remy et al., 2018a; Tung et al., 2022). As WB gains recognition as an ideal product for hemostatic resuscitation, ensuring its safety and functional integrity during storage has become a critical challenge. Current standards recommend leukocyte reduction before storage to reduce risks associated with donor leukocytes and residual cytokines (Chang et al., 2018; Dejigov Monteiro da Silva et al., 2024). However, while immunologic safety has been extensively discussed, the hemostatic consequences of LR in cold-stored WB remain insufficiently defined.

Importantly, standard LR filters (primarily designed for RBCs) do not preserve platelets, which are essential mediators of primary hemostasis and thrombin generation (Morris et al., 2019). Platelet-sparing LR filters exist but are manufactured by a single company, raising concerns about cost, availability, and supply-chain resilience, especially in military or resource-limited settings (Yazer et al., 2021; Smith et al., 2023). Consequently, many centers still use standard LR filters despite the uncertain impact on the hemostatic potency of stored WB.

The previous studies have focused on unfiltered WB, which largely preserves coagulation function during the first 14–21 days, or on WB processed with platelet-sparing LR filters, which maintains functional integrity up to 21 days (Jobes et al., 2011; Haddaway et al., 2019). However, the hemostatic function of WB processed with standard non-platelet-sparing filters (widely used in routine practice) remains largely uncharacterized throughout the entire storage period.

To address this gap, we systematically compared WB processed with a standard filter against matched unfiltered controls. Hematologic, metabolic, and viscoelastic parameters were monitored over 35 days of storage, and to explore potential pathways and key parameters by which LR alters platelet preservation and clotting function. Our findings are intended to provide preliminary experimental evidence that may help refine and inform future whole-blood transfusion strategies.

## 2 Materials and methods

### 2.1 Study design and sample processing

Ten bags of WB (400 mL ± 10%) were collected from healthy male donors under an approved institutional standard operating procedure. All donors were blood group B. Each bag was drawn into a citrate phosphate dextrose adenine (CPDA) collection bag made of polyvinyl chloride (PVC; Shuangwei, Nanjing, China). Within 6 h after WB collection, each bag was aseptically divided into two equal satellite bags (approximately 200 mL each) using a sterile docking device, resulting in 10 genetically matched pairs. One bag from each pair was leukoreduced using a standard non-platelet-sparing filter (Shuangwei, Nanjing, China) by gravity flow from a 60 cm head over 10 min, constituting the leukoreduced whole blood group (LR-WB, n = 10). The matched control bags underwent sealing procedures without filtration, representing the nonleukoreduced whole blood group (NLR-WB, n = 10). This paired design minimizes inter-donor variability and allows for direct assessment of the LR effect. All blood bags were stored at (4 ± 2) °C in a blood bank refrigerator for 35 days after passing routine quality control testing.

Storage-lesion testing was performed on days 0, 7, 14, 21, 28, and 35 from the day of blood collection (Day 0, baseline; room temperature). On each sampling day, the blood bags were gently inverted to ensure homogeneity before sampling. A 12 mL aliquot of WB was obtained using a sterile 15 mL syringe. An overview of the *ex vivo* WB leukoreduction and storage experiment was shown in Supplementary Figure S1.

### 2.2 Hematological and biochemical measurements

#### 2.2.1 RBCs storage damage assessment

WB aliquots were analyzed for complete blood count using a Sysmex XS-1000i hematology analyzer (Sysmex, Japan). RBC membrane stability was evaluated by osmotic fragility testing using graded sodium chloride (NaCl) solutions. After centrifugation (3,600 rpm, 10 min), supernatant absorbance at 540 nm was measured to assess RBC susceptibility to hypotonic stress. The degree of RBC hemolysis in 0.56% NaCl solution was used to calculate the hemolysis increment, which was subsequently applied to derive the EC50 value. Intracellular ATP content was measured by Enzyme-Linked Immunosorbent Assay (ELISA) following boiling lysis (1:4 with ddH_2_O, 10 min), and expressed as μmol/g Hb (ATP kit, Sigma, America). RBC membrane apoptosis was assessed by Aisen Gene D2040 flow cytometry (Aisen Gene, China), Annexin V (BD Biosciences, America) for phosphatidylserine (PS) exposure. Results were expressed as percent positivity. Free hemoglobin (FHb) was quantified from plasma samples using a commercial assay kit (Ruierda Biotechnology, China). The 2,3-diphosphoglycerate (2,3-DPG) level was determined using a human ELISA kit (Anoric Biotechnology, China). All according to the manufacturer’s protocols.

#### 2.2.2 Plasma functions and metabolic changes assessment

After centrifugation at 3,000 rpm for 10 min, plasma was collected for standard coagulation assays using a Werfen ACL TOP coagulometer (Werfen, Spain) and commercial factor assay kits (Tianjin Anoric Biotechnology, China). These parameters included activated partial thromboplastin time (APTT), prothrombin time (PT), thrombin time (TT), fibrinogen (Fib), coagulation factor VIII (FVIII), coagulation factor X (FX), and coagulation factor XIII (FXIII). Supernatants were assessed for routine biochemical indicators using a Beckman DxC 800 Pro biochemical analyzer (Beckman, America). All instruments were calibrated daily according to the manufacturer’s instructions. These parameters included albumin (ALB), globulin (GLB), carbon dioxide (CO_2_), lactate (Lac), potassium (K^+^), sodium (Na^+^), calcium (Ca^2+^), glucose (GLU), and lactate dehydrogenase (LDH). All assays were performed in triplicate. Plasma samples were pretreated or diluted according to the operating guidelines of each instrument.

#### 2.2.3 PLT clot kinetics assessment

Coagulation kinetics were assessed separately using a Haemonetics 5,000 thromboelastograph system (TEG, Haemonetics, America). For each test, 1 mL of WB was transferred into a polyethylene tube preloaded with 40 μL of kaolin and gently inverted to mix. Subsequently, 340 μL of the kaolin-activated sample was pipetted into a disposable TEG cup containing 20 μL of calcium chloride, and the analysis was initiated immediately. These parameters included reaction time (R), kinetics time (K), Angle, and maximum amplitude (MA).

### 2.3 Statistical analysis

The primary analyses focused on describing the *ex vivo* effects of LR and storage duration on WB laboratory and hemostatic parameters. The normality of continuous variables was assessed using the Shapiro–Wilk test. Normally distributed data are presented as mean ± standard deviation (SD) and compared using paired or independent two-tailed t tests, or two-way repeated-measures ANOVA with Šídák’s multiple-comparison correction. Non-normally distributed variables were summarized as median (interquartile range, IQR) and analyzed using Mann–Whitney or Friedman tests with Dunn’s *post hoc* correction. All analyses were performed in R (version 4.3.2); graphs were generated with the ggplot2 package, and final figures were edited in Adobe Illustrator. A two-sided α of less than 0.05 was considered statistically significant.

### 2.4 Exploratory multivariable analyses (WGCNA, mediation, bayesian networks and machine learning)

In addition to the primary statistical analyses, we conducted a series of exploratory multivariable analyses on the experimental dataset. These analyses aimed to characterize higher-order covariation patterns and identify potential pathways and parameters. They were restricted to a single *ex vivo* whole-blood cohort without external validation or replication, and therefore should be regarded as hypothesis-generating rather than confirmatory.

#### 2.4.1 Weighted gene co-expression network analysis (WGCNA)

As an exploratory tool to describe higher-order co-variation patterns within the experimental dataset, we applied WGCNA to the full panel of 31 laboratory indicators. Briefly, standard WGCNA procedures were implemented to construct a topological overlap matrix, identify color-coded co-variation modules, and represent each module by its module eigenscore; module–trait correlations with storage duration and LR status were subsequently analyzed descriptively.

#### 2.4.2 Bayesian network and mediation analysis

To explore whether the observed associations of storage duration or LR status with clot strength (MA) might involve intermediary laboratory parameters, we used a causal mediation analysis framework in R under standard causal assumptions. The potential mediators, such as platelet parameters, K, Angle, Fib, and selected electrolytes, were pre-specified based on biological plausibility and preliminary correlation analysis. For each exposure–outcome pair, we estimated the total, direct, and indirect (mediated) effects. These quantities should be interpreted as model-based estimates within this experimental dataset rather than as definitive proof of causal pathways. In parallel, Bayesian network analysis was applied using the bnlearn and igraph packages in R to explore probabilistic dependencies among storage duration, LR status, laboratory parameters and MA. Network structures were learned from the data and visualized as directed graphs. Given the limited sample size and single-cohort design, both the mediation and Bayesian network results are exploratory and hypothesis-generating only.

#### 2.4.3 Least absolute shrinkage and selection operator (LASSO) based feature selection

As an additional exploratory tool, we employed penalized regression to rank laboratory variables by their association with clot strength (MA) in the experimental dataset. All 31 laboratory variables were considered candidate predictors, with thromboelastographic MA defined as the target outcome. Prior to modeling, all variables were standardized, and occasional missing values were imputed using within-group medians. Feature selection was performed using a regularized linear regression model (least absolute shrinkage and selection operator, LASSO). The optimal penalty parameter (λ) was determined through repeated cross-validation, and model performance was evaluated using mean squared error (MSE). Feature importance was extracted from all models, and variables consistently ranked among the top contributors were identified as candidate predictors. All procedures were implemented using standard open-source workflows in R.

These penalized regression models were used solely for internal feature ranking within the *ex vivo* experimental dataset. No external or independent validation was performed, and the resulting models are not intended to serve as clinically deployable prediction tools.

## 3 Result

### 3.1 Effects of LR status and storage time on platelet/viscoelastic indices

**FIGURE 1:**
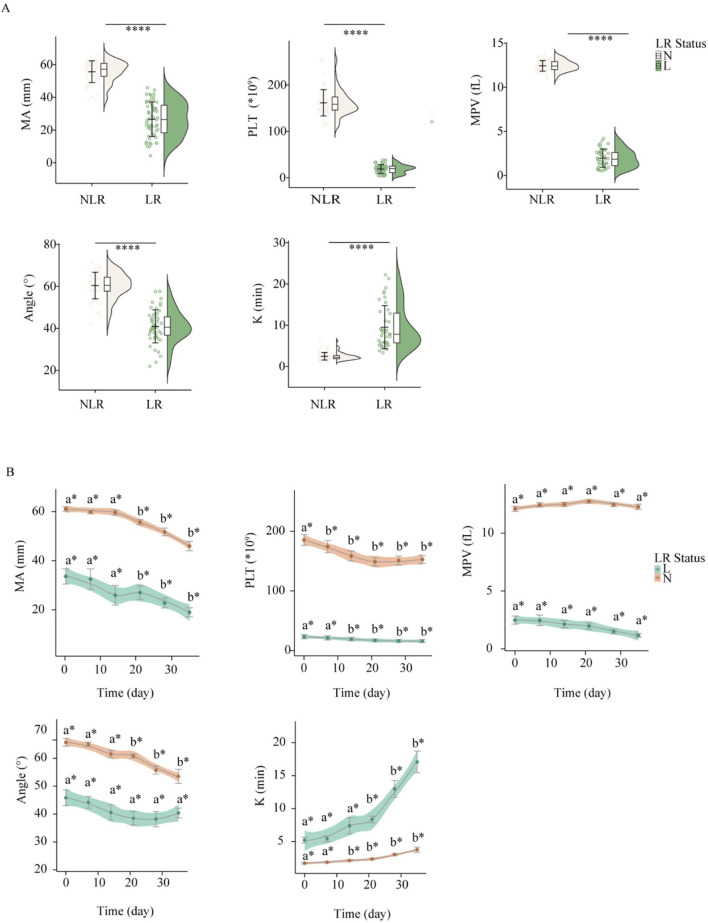
Effects of leukoreduction (LR) status and storage duration on hemostatic parameters. **(A)** Baseline comparisons (Day 0) between LR-WB and NLR-WB, shown as box-and-violin plots. *P < 0.05, **P < 0.01, ***P < 0.001, ****P < 0.0001 indicate statistically significant differences compared to the control group. **(B)** Temporal changes of parameters during storage (Day 0, 7, 14, 21, 28, 35) in LR-WB and NLR-WB, shown as line plots with mean ± SD. * indicates a significant between-group difference at the same time point (*P* < 0.05). Within each group, Day 0 is labeled “a”; other time points sharing “a” do not differ from Day 0, whereas “b” indicates a significant difference vs. Day 0 (*P* < 0.05). See Abbreviations for full definitions.

Focusing on platelet-related and viscoelastic indices, we analyzed both baseline differences and storage-related trajectories between LR-WB and NLR-WB (Figure 1). On Day 0 (immediately after leukoreduction), LR-WB showed markedly lower platelet count than paired NLR-WB (23.40 ± 2.00 vs. 185.20 ± 2.93 *10^9^/L, *p* < 0.05). Consistently, viscoelastic clot strength was reduced, with lower MA (33.6 ± 9.82 vs. 61.09 ± 2.97 mm), prolonged K (5.20 ± 1.37 vs. 1.70 ± 0.35 min), and lower Angle (45.83 ± 8.82 vs. 65.68° ± 4.33°) (all *p* < 0.05). During storage, both LR-WB and NLR-WB showed consistent temporal trends: platelet, MPV, MA, and Angle declined progressively, whereas K increased (Figure 1B). In addition to these time-dependent effects, LR-WB and NLR-WB maintained parallel but separated trajectories. These group differences persisted throughout storage and, for several parameters, became more pronounced at later time points. Overall, LR exerted both immediate and sustained effects on platelet- and viscoelastic-related indices, beyond the time-dependent changes observed during storage.

### 3.2 Global trends of laboratory indicators during 35-day storage of WB

**FIGURE 2:**
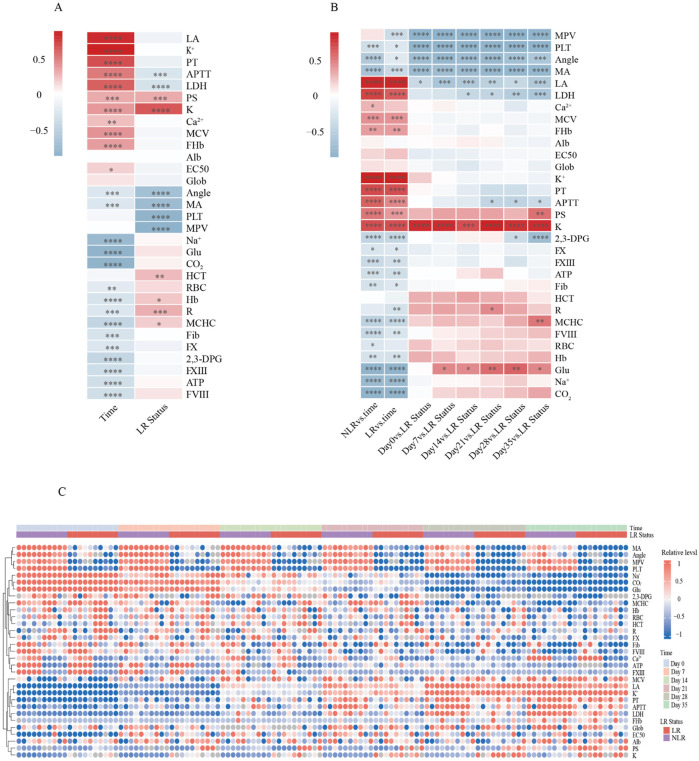
Descriptive heatmap overview of changes in measured parameters across leukoreduction (LR) status and storage duration in the *ex vivo* whole blood (WB) experiment. All indicator values were standardized (z-score transformation) before plotting. Deeper colors represent stronger correlations in all panels. **(A)** Summary heatmap showing the overall effects of storage time and LR status on all measured parameters. Blue indicates a positive correlation and red indicates a negative correlation relative to the reference condition. **(B)** Heatmap showing the association of storage time with parameters in the NLR group, and the combined effects of storage duration and LR status, highlighting relationships between each storage time point and group (LR vs. NLR). Blue indicates a positive correlation and red indicates a negative correlation relative to the reference condition. **(C)** Integrated heatmap of all individual samples across six storage time points (Day 0, 7, 14, 21, 28, and 35) and both groups (LR and NLR). Each dot represents the relative value of a parameter for a single sample, with variables along the y-axis hierarchically clustered. Red indicates a positive correlation and blue indicates a negative correlation. This heatmap was used as a descriptive visualization to explore patterns within the experimental dataset and was not treated as an independent discovery or validation analysis. See Abbreviations for full definitions.

To examine temporal changes in LR-WB and NLR-WB, we generated a descriptive heatmap with hierarchical clustering to visualize similarity among variables (Figure 2). Two main axes of variation emerged: storage duration (Days 0–35) and processing method (LR vs. NLR).

Over time, the platelet-related and viscoelastic parameters (including PLT count, K, and MA) showed progressive and significant adverse changes consistent with platelet damage (*p* < 0.05). By contrast, the metabolic markers such as lactate, LDH, FHb, and K^+^ increased steadily, whereas most coagulation factors declined modestly. Figure 2B (rightmost columns) shows LR-NLR comparisons at each time point. Across storage, LR-WB consistently exhibited lower PLT count, MPV, MA, and Angle compared with NLR-WB (*p* < 0.05).

Hierarchical clustering grouped variables into two primary clusters: platelet/viscoelastic parameters and coagulation factors, and metabolic and electrolyte indicators. These clusters remained stable throughout storage, indicating similar temporal change patterns within each group.

### 3.3 Exploratory WGCNA identifies distinct parameter modules linked to storage time and LR status

**FIGURE 3:**
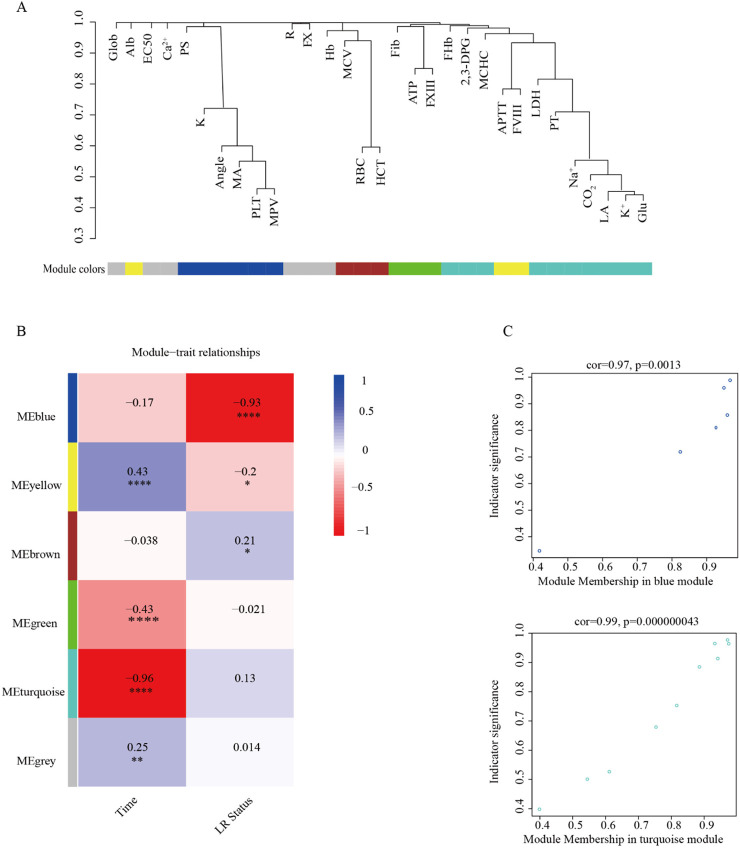
Exploratory weighted gene co-expression network analysis (WGCNA) of storage-related parameters the *ex vivo* whole blood (WB) experiment. **(A)** Hierarchical clustering dendrogram of all measured parameters based on topological overlap, with modules assigned different colors. The gray module contains unassigned parameters. **(B)** Module–trait correlation heatmap showing Pearson correlation coefficients (r) between each module and two traits (storage duration and LR status). Numbers in each cell indicate correlation coefficients with corresponding p-value (*P < 0.05, **P < 0.01, ***P < 0.001). Color intensity reflects the magnitude and direction of correlation (blue, positive; red, negative). **(C)** Distribution of representative parameters within key modules. The turquoise module was strongly negatively correlated with storage duration and enriched in metabolic indicators, while the blue module was negatively correlated with LR status and enriched in platelet function–related parameters. These WGCNA results are exploratory, were derived from a single *ex vivo* experimental dataset without external replication, and are intended to generate hypotheses about parameter co-variation rather than to define validated modules or biomarkers. See Abbreviations for full definitions.

WGCNA identified six distinct co-variation modules, and each labeled with a unique color (Figure 3). Module sizes ranged from 3 to 10 parameters, with the gray module comprising variables that were not assigned to any specific cluster. Module–trait correlation analysis showed a strong negative association between the turquoise module and storage duration (*r* = −0.96, adjusted *p* < 0.0001). The blue module was negatively associated with LR status (*r* = −0.93, adjusted *p* < 0.0001). Metabolic indicators were predominantly located in the turquoise module, whereas platelet function related parameters were enriched in the blue module.

### 3.4 Mediation and bayesian network analysis of LR and storage effects on MA

**FIGURE 4:**
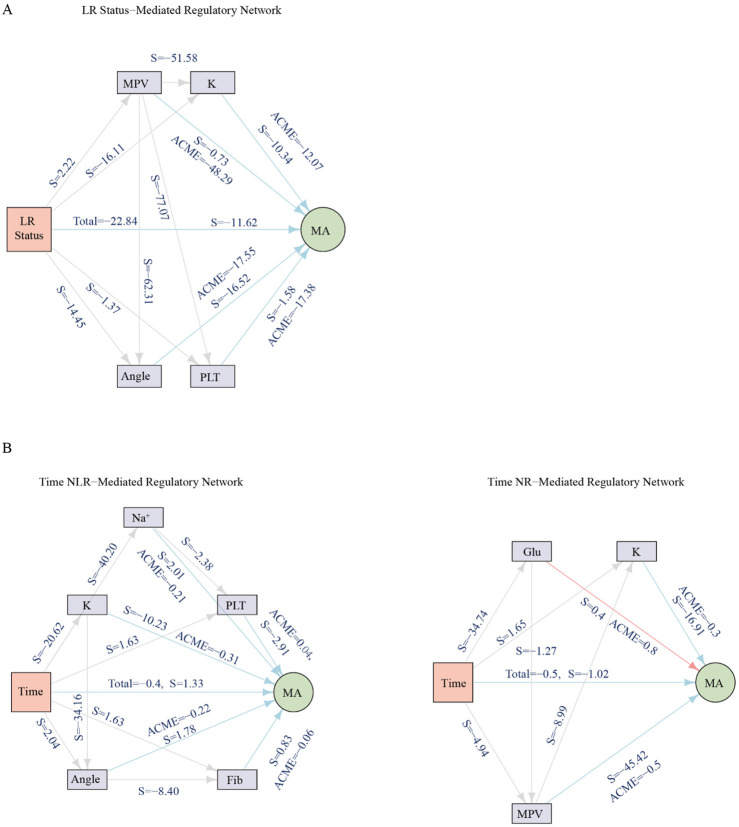
Exploratory mediation analysis and Bayesian network models to characterize potential pathways associating leukoreduction (LR), storage duration, and clot strength (MA) in the experimental dataset. **(A)** Exploratory mediation analysis of LR status on maximum amplitude (MA). The total effect was partitioned into direct and indirect effects through candidate mediators, including platelet count, mean platelet volume (MPV), kinetics time, angle, fibrinogen, and glucose. **(B)** Exploratory mediation analysis of storage duration on MA trajectories in LR-WB and NLR-WB. Indirect effects (average causal mediation effect, ACME) quantify the contribution of each mediator, while direct effects (average direct effect, ADE) represent residual effects after accounting for mediators. Edge strength (S) was reported for each arc in the Bayesian network, representing the signed magnitude of the inferred parent–child dependency; larger absolute values correspond to stronger associations, and the sign indicates directionality. These mediation and network models are exploratory, were fitted on the single *ex vivo* experimental dataset without external validation, and should be interpreted as hypothesis-generating only. See Abbreviations for full definitions.

To further explore potential mechanisms underlying the observed decline in MA over storage, we performed exploratory mediation and Bayesian network analyses with MA as the outcome (Figure 4; Supplementary Table S1). LR was associated with a significant reduction in MA in the mediation model (total effect β = −22.84, *p* = 0.002). In these model-based analyses, this association appeared to be partly linked to platelet- and clot-kinetics pathways. MPV was identified as a potential mediator, whereas Angle, platelet count, and K also exhibited indirect associations within the modeled framework. Thus, within this experimental dataset, the LR-MA association may be related to platelet indices (PLT and MPV) and clot formation dynamics (Angle, K).

Storage duration exerted a progressive negative association on MA in both LR and NLR groups. In the LR-WB, this decline was statistically linked to MPV and K; whereas in the NLR-WB, it was associated with broader pathways, including K and Angle. Glucose showed a suppression pattern, with its indirect effect opposing the overall decline and modestly attenuating the reduction in MA.

### 3.5 Exploratory machine-learning feature ranking of determinants of MA

**FIGURE 5:**
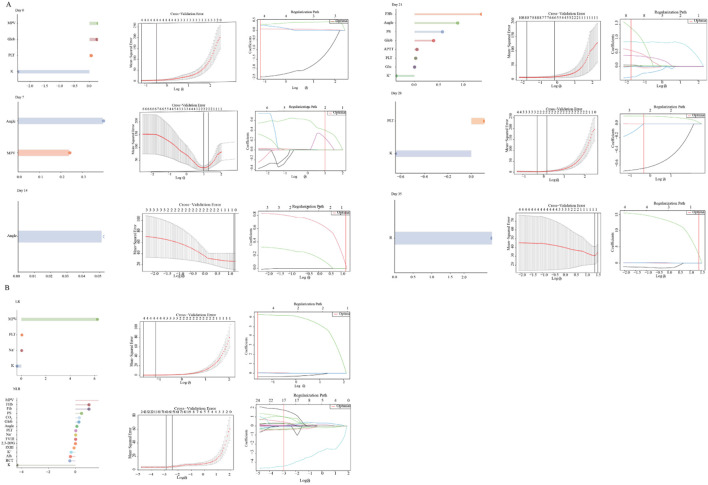
Exploratory machine learning-based feature ranking of variables linked to clot strength (MA) in the experimental dataset. **(A)** Overview of predictors at each storage time point (Day 0, 7, 14, 21, 28, and 35) based on penalized regression models fitted within the *ex vivo* whole-blood dataset. **(B)** Example of the internal feature selection process stratified by LR status. From left to right: ranked coefficients of selected predictors, cross-validation error curves across penalty parameters (log λ), and corresponding regularization paths. These analyses were used as exploratory, internal feature-ranking tools within the experimental dataset, were not externally validated, and are not intended for clinical prediction or deployment. See Abbreviations for full definitions.

To explore model-based predictors of MA beyond predefined mediators, we applied LASSO regression as an exploratory feature-ranking tool, stratified by storage time (Figure 5A) and LR status (Figure 5B). Overall, in early storage, MA ranking was mainly driven by platelet-related indices and clot-kinetics parameters. In contrast, during later storage, hemolysis- and RBC injury–related features (FHb and PS exposure) became more prominent within this experimental dataset. Stratified analyses indicated that LR-WB showed a relatively simpler feature pattern, largely dominated by platelet indices and clot kinetics. By comparison, NLR-WB demonstrated a broader feature profile, including variables related to hemolysis, metabolism, and coagulation (Figure 5).

## 4 Discussion

The impact of standard LR, widely applied for immunologic safety, on the hemostatic integrity of stored WB remains underexplored. The previous studies on WB storage have mainly focused on NLR-WB or those processed with platelet-sparing filters, and most have been descriptive in nature (Haddaway et al., 2019; Assen et al., 2020; Susila et al., 2023; Tan et al., 2025). However, the filters commonly used in civilian and military settings are not platelet-sparing, and their effects on the coagulation potential of WB during prolonged storage remain poorly characterized. To address this gap, we conducted an *ex vivo* 35-day cold storage study comparing standard LR-processed WB with matched unfiltered controls, evaluating metabolic, platelet function, and viscoelastic parameters. By further integrating exploratory mediation analysis and machine learning-based feature ranking using this experimental dataset, we investigated potential pathways through which LR and storage duration synergistically contribute to hemostatic impairment. This combined experimental and analytical framework sets our study apart from prior work, providing detailed time-course data and hypothesis-generating insights into coagulation disturbances associated with prolonged WB storage.

The global descriptive heatmap and exploratory WGCNA analyses suggested that storage-related indices within this experimental dataset followed two coordinated temporal trajectories rather than purely random variation. The platelet–clot elasticity axis of variables (PLT, MPV, MA, and Angle) exhibited a progressive decline, whereas the metabolic–hemolytic–electrolyte axis (lactate, LDH, FHb, and K^+^) showed a gradual increase. This interpretation is supported by prior studies showing that storage-related changes in membrane permeability and ion homeostasis are associated with progressive increases in K^+^, LDH, FHb, and lactate levels during WB storage (Flatt et al., 2014; Mykhailova et al., 2020).

The longitudinal trajectory analysis showed that LR-WB and NLR-WB followed parallel but progressively divergent storage patterns, suggesting that LR predominantly compromises baseline hemostatic capacity, rather than substantially altering the overall trajectory of storage-related deterioration in this *ex vivo* model. These effects may be likely related to early platelet depletion during filtration. Previous studies have shown that activated platelets expressing P-selectin form aggregates with leukocytes and release platelet-derived microparticles (PMPs), which are captured together with leukocytes by the filter matrix (Forlow et al., 2000; Remy et al., 2018b).

The reduced number of platelets reserved at baseline, which may leave LR units more vulnerable to subsequent viscoelastic impairment and help explain the wider separation in MA and Angle over time in this *ex vivo* model. In addition, prior studies have indicated that filter design, pore size, whether devices are platelet-sparing, and the timing of LR all substantially influence platelet retention and clot stability (Haddaway et al., 2019; Morris et al., 2019; Rice et al., 2021). Together, these findings suggest that LR-WB is not a single, interchangeable product in terms of hemostasis. The leukoreduction method and workflow can strongly affect the platelet-dependent clot strength delivered by one unit.

In practice, LTOWB has been used as the initial transfusion product in acute perinatal/obstetric hemorrhage (Bahr et al., 2019; Carr et al., 2022; Park et al., 2025). It can provide RBCs, plasma factors, and platelets at the same time, rather than stepwise component transfusion. In a recent perinatal series, LTOWB was used first, and all patients later received additional blood products after initial stabilization (Carr et al., 2024). This “front-line” use makes the hemostatic profile at the time of transfusion clinically relevant, including very early storage. As reported in current practice, some programs elect to transfuse LTOWB within 7–14 days and convert unused units to packed RBCs after day 14; platelet-sparing leukoreduction filters are used in some settings (Carr et al., 2022; Yazer et al., 2024; Ghaedi et al., 2025). In this context, our *ex vivo* findings suggest that standard (non–platelet-sparing) leukoreduction can cause an immediate decrement in platelet availability and viscoelastic clot strength at Day 0, which may reduce the “front-line” hemostatic capacity delivered by a unit. These implications should be viewed as product-characterization evidence. They support direct comparisons of leukoreduction approaches and prospective clinical evaluation, rather than definitive practice recommendations.

Global and longitudinal analyses provided a descriptive framework for how LR and storage jointly relate to hemostatic deterioration in this *ex vivo* model. However, they did not by themselves identify the pathways primarily associated with MA decline. Given that LR disproportionately affects platelet-related variables, while storage time drives cumulative metabolic and hemolytic stress, we hypothesized that these effects might act through distinct yet potentially converging mechanisms. To further explore and separate these contributions, we conducted parallel mediation analyses to decompose the total associations of LR and storage period with clot strength (MA) into model-based indirect components through platelet metrics and metabolic alterations, and residual direct components within this experimental dataset.

The first pathway involves platelet reserve and function. Under LR conditions, reductions in platelet count and MPV showed statistically significantly mediation of the observed decline in MA in our exploratory mediation models. This is consistent with the physiology that MA primarily reflects platelet-fibrin interactions (Peng et al., 2018). Cold storage can trigger ADAM17-mediated shedding of GPIbα and downregulation of GPVI, both of which may weaken collagen-dependent platelet adhesion and activation (Montague et al., 2018; Miles et al., 2021). Additionally, falling ATP levels during storage lead to changes in the cell membrane that cause PS to move to the outside of the platelet surface (Shapira et al., 2000; Lai et al., 2002). This may increase procoagulant activity but also reduces platelet function by lowering GPIIb/IIIa activity and possibly promoting the loss of GPIbα and GPVI, which together may contribute to the decline in MA (Shapira et al., 2000; Lai et al., 2002). The second pathway relates to clot kinetics. The prolonged K and reduced Angle emerged as statistically significant mediators of the associations of LR and storage duration with MA in our mediation analyses. The fibrinogen levels were similar between LR-WB and NLR-WB, which suggests that LR does not lower fibrinogen levels. However, LR-WB had a longer K and a smaller Angle, suggesting slower fibrin-based clot growth. These observations indicate that the impairment may relate more to clot quality than to the absolute amount of fibrinogen. Some studies, however, report different structural findings under 4 °C storage: clots formed from cold-stored platelets can have thinner and straighter fibers, more branch points, and stronger FXIII-mediated cross-linking, resulting in denser and mechanically stronger fibrin networks (Nair et al., 2017; Nair et al., 2021). These differences may be explained by variations in study design, such as platelet activation, FXIII availability, and whether clot contraction is preserved during testing. In our study, prolonged storage may still reduce FVIII and FXIII activity in both groups, slowing fibrin build-up, while LR could add an extra effect by removing leukocyte-derived antifibrinolytic factors and weakening platelet–fibrin bridging through GPIIb/IIIa (Matveeva et al., 2022). Together, these effects may help explain why LR units in our experiment showed earlier and stronger impairment in clot propagation despite similar fibrinogen levels.

In exploratory machine-learning analyses applied within this experimental dataset, we observed patterns suggesting that storage lesion pathways evolve over time. The platelet-driven clotting predominated in the early phase, while injury to RBC membranes and metabolic issues appeared to become more significant during prolonged storage. The late-phase selection of RBCs surface PS and FHb as candidate predictors of MA may suggest that RBC membrane stability may be important for maintaining clot strength, although these findings are hypothesis-generating and require independent confirmation. Flexible RBCs can raise clot viscoelasticity and fit into and tighten the fibrin network (Gersh et al., 2009). However, the decreased ATP and 2,3-DPG and damaged membranes lower RBCs deformability and weaken this mechanical support, leading to a lower MA (Gersh et al., 2009; Yoshida et al., 2019). RBC-fibrinogen interactions can increase stiffness under favorable conditions, while this benefit is blunted when RBC membranes are unstable, yielding more heterogeneous networks and less net strength (Guedes et al., 2018; Alkarithi et al., 2025). Taken together, these exploratory findings raise RBC membrane stability and energy preservation as potential targets for improving WB storage quality and for guiding the design of future transfusion studies, rather than providing definitive evidence to change current practice.

LR has been reported to reduce proinflammatory cytokine release and microparticle generation, which may help explain the simplified feature profile observed in LR-WB in our exploratory models (Richter et al., 2018; Silva et al., 2025). In contrast, NLR-WB displayed a more complex signature involving hemolysis, metabolic disturbances, and coagulation protein depletion, which may be linked to residual leukocyte-driven amplification of storage-related injury. These findings suggest that LR may not only limit immune activation but also influence the dominant pathways contributing to storage lesions in this *ex vivo* setting. Importantly, predictors identified by machine learning in this study indicate associations within the experimental dataset rather than causality. These relationships require confirmation through prospective functional assays and clinical studies. Future work combining machine learning with multi-omics data and patient-centered endpoints could further facilitate the development of a comprehensive predictive framework for assessing WB quality and optimizing storage strategies.

Several limitations should be noted. This was an *ex vivo*, paired-unit study with a limited number of whole-blood units; therefore, the findings characterize product-level changes and cannot be directly extrapolated to patient outcomes. We evaluated a single leukoreduction workflow (a standard, non–platelet-sparing filter applied under a specific protocol), so applicability to platelet-sparing filters, alternative devices, or different leukoreduction timing remains uncertain. In addition, WGCNA and the mediation/Bayesian network and LASSO analyses were exploratory and model-based, and should be interpreted as hypothesis-generating rather than causal or broadly generalizable. Finally, we did not define clinical “acceptability” thresholds *a priori*; the 7–14-day transfusion window referenced in some programs reflects prior practice rather than a recommendation derived from our dataset.

## 5 Conclusion

In summary, LR was associated, in this *ex vivo* model, with reduced early platelet reserve and may contribute to accelerated viscoelastic decline during storage, whereas storage duration itself appeared to drive cumulative metabolic stress and potential fibrin-related kinetic impairment. These dual processes may help explain the observed divergence of LR-WB and NLR-WB trajectories and highlight the need to balance LR with preservation of hemostatic function. From a translational perspective, these findings suggest that platelet-related and RBC injury–related indices may serve as potential quality indicators for monitoring stored WB and guiding blood inventory management, although these applications require validation in clinical settings. Future studies integrating multi-platform assays and clinical transfusion outcomes are warranted to better define the impact of LR on storage quality and optimize LR strategies.

## Data Availability

The datasets presented in this article are not readily available because the data that support the findings of this study are available on request from the corresponding authors, upon reasonable request. Requests to access the datasets should be directed to Lei Liu, liulei890207@163.com.
