# Supplementary material for: Effects of leukoreduction and storage duration on whole blood hemostatic function: a prospective ex vivo observational study
Source: Front Cell Dev Biol. 2026 Mar 27;14:1765877. doi: 10.3389/fcell.2026.1765877 (PMC13066190; doi:10.3389/fcell.2026.1765877)
Supplement: Supplementary file 1 [file DataSheet1.doc]

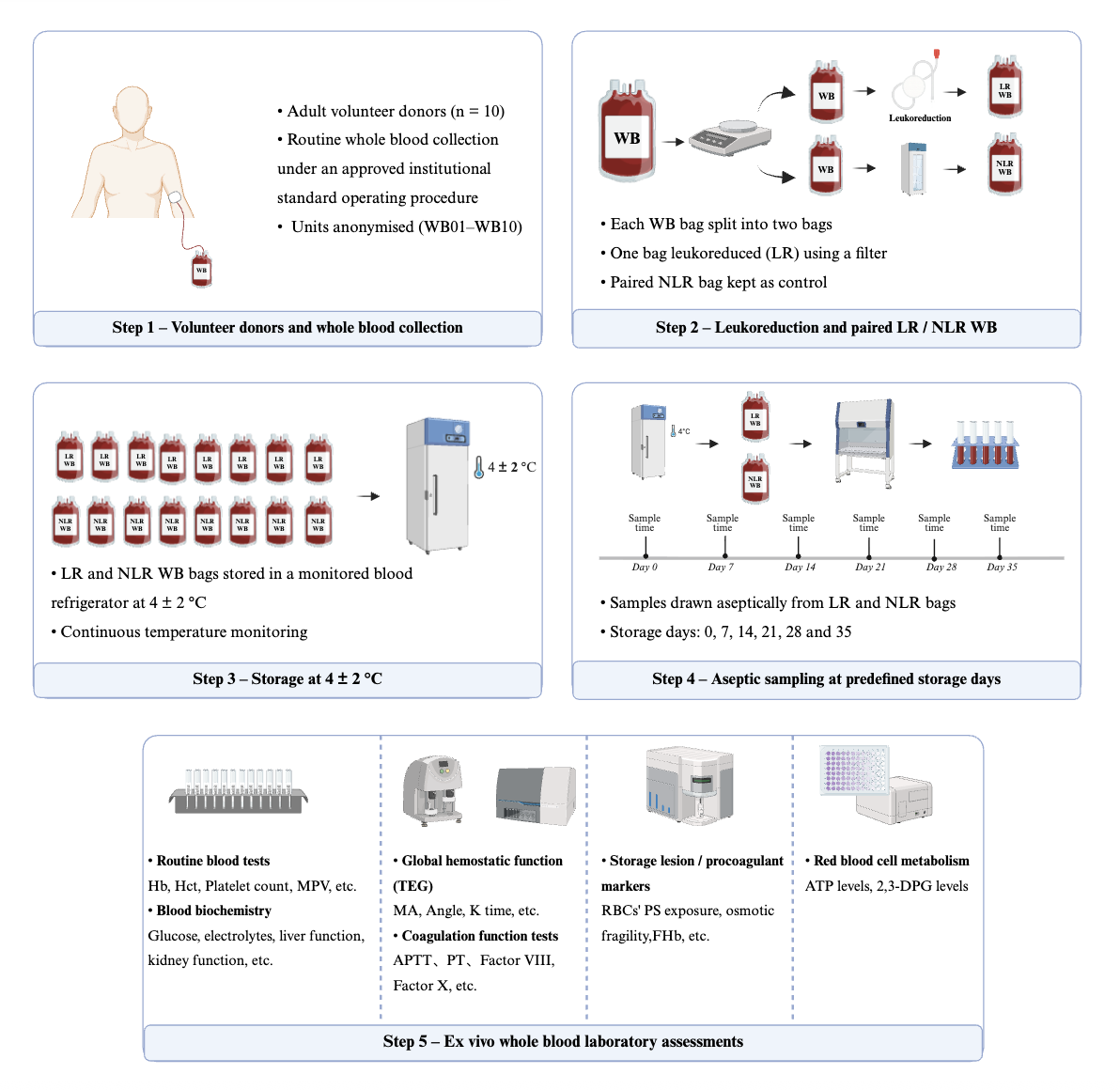


**Supplementary Figure 1. Workflow of the ex vivo whole blood (WB) leukoreduction (LR) and storage experiment.** Ten WB units from adult volunteer donors were collected and anonymized (WB01–WB10). Each bag was split into two bags, one of which underwent LR using a filter, while the paired bag remained NLR as a control. LR and NLR WB bags were stored in a monitored blood refrigerator at 4 ± 2 °C. Aseptic samples were drawn from each bag at predefined storage days (0, 7, 14, 21, 28 and 35). WB samples were used for routine laboratory tests (e.g. hemoglobin, hematocrit, platelet count), global hemostatic assessment by thromboelastography (TEG: MA, Angle, K time), and exploratory markers of storage lesion such as RBCs’ phosphatidylserine exposure.

**Supplementary Table 1. Mediation analysis results for the effect of leukoreduction (LR) status and time on clot strength (MA)**

| **Group** | **from** | **to** | **strength** | **ACME** | ***P*-value** | **Direct** | **Total** | **Prop** | **adjusted *P*-value** |
| --- | --- | --- | --- | --- | --- | --- | --- | --- | --- |
| LR Status | LR Status | MPV | 2.22 | NA | NA | NA | -22.84 | NA | NA |
| LR Status | LR Status | Angle | -14.45 | NA | NA | NA | -22.84 | NA | NA |
| LR Status | LR Status | PLT | -1.37 | NA | NA | NA | -22.84 | NA | NA |
| LR Status | LR Status | K | -16.11 | NA | NA | NA | -22.84 | NA | NA |
| LR Status | LR Status | MA | -11.62 | -22.84 | NA | NA | -22.84 | NA | NA |
| LR Status | MPV | MA | -0.73 | -48.29 | 0.00 | 25.45 | -22.84 | 2.11 | 0.00 |
| LR Status | Angle | MA | -16.52 | -17.56 | 0.00 | -5.28 | -22.84 | 0.77 | 0.00 |
| LR Status | PLT | MA | -1.58 | -17.39 | 0.00 | -5.45 | -22.84 | 0.76 | 0.00 |
| LR Status | K | MA | -10.34 | -12.07 | 0.00 | -10.77 | -22.84 | 0.53 | 0.00 |
| LR Status | MPV | PLT | -77.07 | NA | 0.00 | 25.45 | -22.84 | 2.11 | 0.00 |
| LR Status | MPV | Angle | -62.31 | NA | 0.00 | 25.45 | -22.84 | 2.11 | 0.00 |
| LR Status | MPV | K | -51.58 | NA | 0.00 | 25.45 | -22.84 | 2.11 | 0.00 |
| LR-time | Time | Glucose | -34.74 | NA | NA | NA | -0.52 | NA | NA |
| LR-time | Time | MPV | -4.94 | NA | NA | NA | -0.52 | NA | NA |
| LR-time | Time | K | 1.65 | NA | NA | NA | -0.52 | NA | NA |
| LR-time | Time | MA | -1.02 | -0.52 | NA | NA | -0.52 | NA | NA |
| LR-time | Glucose | MA | 0.40 | 0.80 | 0.01 | -1.33 | -0.52 | -1.54 | 0.03 |
| LR-time | MPV | MA | -45.42 | -0.49 | 0.00 | -0.03 | -0.52 | 0.94 | 0.00 |
| LR-time | K | MA | -16.91 | -0.33 | 0.00 | -0.19 | -0.52 | 0.63 | 0.00 |
| LR-time | MPV | K | -8.99 | NA | 0.00 | -0.03 | -0.52 | 0.94 | 0.00 |
| LR-time | Glucose | MPV | -1.27 | NA | 0.01 | -1.33 | -0.52 | -1.54 | 0.03 |
| NLR-time | Time | K | -20.62 | NA | NA | NA | -0.43 | NA | NA |
| NLR-time | Time | Angle | 2.04 | NA | NA | NA | -0.43 | NA | NA |
| NLR-time | Time | Na^+^ | -40.20 | NA | NA | NA | -0.43 | NA | NA |
| NLR-time | Time | Fib | 1.63 | NA | NA | NA | -0.43 | NA | NA |
| NLR-time | Time | PLT | 1.63 | NA | NA | NA | -0.43 | NA | NA |
| NLR-time | Time | MA | 1.33 | -0.43 | NA | NA | -0.43 | NA | NA |
| NLR-time | K | MA | -10.23 | -0.31 | 0.00 | -0.12 | -0.43 | 0.71 | 0.00 |
| NLR-time | Angle | MA | 1.78 | -0.22 | 0.00 | -0.21 | -0.43 | 0.51 | 0.00 |
| NLR-time | Na^+^ | MA | 2.01 | -0.21 | 0.00 | -0.22 | -0.43 | 0.49 | 0.02 |
| NLR-time | Fib | MA | 0.83 | -0.06 | 0.01 | -0.37 | -0.43 | 0.15 | 0.03 |
| NLR-time | PLT | MA | -2.91 | -0.04 | 0.01 | -0.39 | -0.43 | 0.10 | 0.04 |
| NLR-time | K | Angle | -34.16 | NA | 0.00 | -0.12 | -0.43 | 0.71 | 0.00 |
| NLR-time | Angle | Fib | -8.40 | NA | 0.00 | -0.21 | -0.43 | 0.51 | 0.00 |
| NLR-time | Na^+^ | PLT | -2.38 | NA | 0.00 | -0.22 | -0.43 | 0.49 | 0.02 |

The table reports estimated edge strength (S), average causal mediation effects (ACME), direct and total effects, proportion mediated (Prop), and corresponding p values. NA = not applicable. See Abbreviations for full definitions.
